# Virtual Reality-Based Intervention for Enhancing Upper Extremity Function in Children With Hemiplegic Cerebral Palsy: A Literature Review

**DOI:** 10.7759/cureus.21693

**Published:** 2022-01-28

**Authors:** Chanan Goyal, Vishnu Vardhan, Waqar Naqvi

**Affiliations:** 1 Pediatric Physiotherapy, Datta Meghe Institute of Medical Sciences, Wardha, IND; 2 Pediatric Physiotherapy, Government Physiotherapy College, Raipur, IND; 3 Cardiorespiratory Physiotherapy, Datta Meghe Institute of Medical Sciences, Wardha, IND; 4 Community Physiotherapy, N.K.P. Salve Institute of Medical Sciences, Nagpur, IND

**Keywords:** hand function, upper extremity function, pediatric physiotherapy, infantile hemiplegia, vr games, unilateral cp, hemiplegic cerebral palsy, serious games, virtual reality

## Abstract

Cerebral palsy (CP) is the most common cause of motor disability in the pediatric population, with hemiplegia as one of the most widely seen subtypes of spastic CP. Although most of the children with hemiplegic CP are independent ambulators, deficits in hand function of the affected side remain a major concern of caregivers and children themselves. Children use the unaffected upper extremity to compensate for the weakness in the affected one, which consequently leads to the disuse of the hemiparetic upper extremity. Interactive virtual environments can enhance the activation of brain areas during training by providing feedback that can catalyze neuroplastic changes for improved function. Although numerous studies have been conducted on the impact of virtual reality (VR)-based rehabilitation in adults with stroke, studies on its use in the pediatric population are scarce. The three broad categories of VR systems based on the type of human-computer interactions are feedback-focused, gesture-based, and haptic-based. Preliminary studies have shown promising results of VR intervention in improving motor function, including upper extremity function, in children with hemiplegic CP. It is an engaging and entertaining intervention that adds an advantage of high compliance due to motivation. The current literature consists of studies with highly heterogeneous groups of participants and small sample sizes. Further investigation on children with a specific type of CP with advanced VR systems technology is warranted.

## Introduction and background

Cerebral palsy (CP) is the most common cause of motor disability in the pediatric population [1,2]. The worldwide incidence has been reported to be two to three per 1,000 live births [3,4]. However, underdeveloped and developing countries have a much higher incidence of CP compared with developed ones, although specific data on these nations are unavailable. Spastic-type CP is the most common type, comprising 85%-91% of all CP cases. Dyskinetic CP, which includes athetosis and dystonia, comprises 4%-7% of cases, whereas ataxic CP and hypotonic CP constitute 4%-6% and 2%, respectively [2]. Spastic CP is further sub-classified into hemiplegia (38% of all spastic CP cases), diplegia (37%), and quadriplegia (constituting 24%). Thus, out of all the subtypes, hemiplegia is the most widely seen presentation in children with CP [2]. Hemiplegic CP can be attributed to stroke, vascular malformations, unilateral intraventricular hemorrhage, or periventricular leukomalacia in the early stages of brain development [5]. The characteristic features of hemiplegic CP or unilateral CP include unilateral spasticity, asymmetrical alignment, the establishment of handedness before 18 months of age, and differences in functional abilities between the two upper extremities [5]. Although most children with hemiplegic CP walk independently, deficits in hand function remain a major concern of caregivers and of the children themselves [5]. Almost half of the children with hemiplegic CP have more impairment in the upper extremity, particularly with the hands than in the lower extremity [6]. Children with infantile hemiplegia often only use the unaffected upper extremity due to weakness and poor voluntary control in the affected one. This consequently leads to the disuse of the hemiparetic upper extremity [7].

Although numerous studies have evaluated the impact of virtual reality (VR)-based rehabilitation in adults with stroke, investigations in the pediatric population are scarce. VR seems to be an effective intervention for improving motor function in children with CP based on the results of preliminary studies [8,9]. This review attempts to summarize the currently available literature on the effectiveness of VR-based interventions on upper extremity function in children with hemiplegic CP in order to identify gaps in the research and perhaps raise relevant research questions for future investigations in this domain.

## Review

The Medline and Google scholar databases were searched using the keywords “virtual reality,” “VR games,” “hemiplegic cerebral palsy,” “unilateral cerebral palsy,” and “infantile hemiplegia”. For the review, inclusion criteria encompassed studies published in the last two decades, in the English language, that assessed the impact of VR-based therapy on the upper extremity function in children with hemiplegic CP, irrespective of the type of VR system used.

Neuroplasticity and contemporary interventions

Neuroplasticity is considered to be the basis for learning in the intact brain as well as for relearning in the damaged brain that takes place through rehabilitative training. Neuroplasticity requires sufficient repetition and training intensity. The training sessions must be sufficiently striking to induce plasticity. Training-induced plasticity occurs more readily in younger brains. Plasticity in response to training experience can enhance the acquisition of specific skills and behavior [10]. Evidence indicates that goal-directed and activity-based intensive interventions are much more effective compared with the usual care in enhancing upper extremity functional outcomes [11]. Constraint-induced movement therapy (CIMT) and bimanual (BIM) training provide options for such intensive management. However, CIMT has an inconclusive impact on the long-term development of hand function in children with unilateral CP [12]. A study found that CIMT improves unimanual capacity, whereas BIM improves its performance [13]. Nevertheless, the quest for finding a rehabilitative intervention approach that is motivating and engaging for children is a need. VR-based gaming appears to fit in well with the requirement of a high number of repetitions of functional movements within a short period while being highly appealing to children.

VR as intervention

Interactive virtual environments can enhance the activation of brain areas during training by providing feedback that can catalyze neuroplastic changes for improved function [14]. Numerous studies have investigated biofeedback-based interventions for children with CP [15].

VR-based therapy has been used for the rehabilitation of adults with stroke effectively [16,17]. A few studies have investigated the effect of VR intervention in children with CP. In 2021, Jha et al. reported that VR gaming coupled with physiotherapy improved balance (as measured using the Pediatric Balance Scale and Kids-Mini-Balance Evaluation Systems Test) in bilateral CP, but they did not find VR gaming with physiotherapy significantly superior to physiotherapy alone in improving gross motor function (as measured using the Gross Motor Function Measure-88) and routine functioning (as measured using the Wee-Functional Independence Measure). In Jha et al.'s study, each exercise session lasted for 60 minutes, and a single session was conducted every day for four days a week over a period of six weeks [18]. In another recently published study, Avcil et al. conducted a study incorporating 60 minutes of VR intervention for three days per week over a period of eight weeks and concluded that V-based gaming has a superior effect on manual dexterity (as measured using the Minnesota Manual Dexterity Test) in children with CP compared with physiotherapy based on neurodevelopmental treatment. However, this superiority was not established for functional abilities (as measured using the Childhood Health Assessment Questionnaire and Duruoz Hand Index) and grip strength (as measured with a dynamometer) [19]. Nonetheless, Cortez-Perez et al. attributed the improvement in grip strength, as well as gross and fine motor dexterity of the majorly affected upper extremity, in children with unilateral CP to VR-based intervention [20].

VR-based interactive serious games were also effective in the telerehabilitation of children with CP. Children enjoyed the sessions, their performance improved, and the intensity of their physical activity increased [21]. The studies conducted by Golomb et al. documented the positive effects of VR-based telerehabilitation for children with hemiplegic CP, such as improvement in hand function and in forearm bone mineral content (as measured by dual-energy X-ray absorptiometry), and found these gains were maintained even 14 months after the discontinuation of the intervention [22,23]. Furthermore, expansion in brain activation areas with paretic hand use (as measured by functional magnetic resonance imaging), reflecting improved hand function, was noted [22]. These findings agreed with those of You et al., who attributed cortical reorganization to VR-based intervention [24]. Similarly, a study by Roberts et al. [25] published in 2021 investigated the combined impact of CIMT, robotic therapy, and VR games and found improvement in hand function, as well as occupational performance, that persisted even after six months following treatment.

Motor learning principles like variable practice and enhanced feedback are suitably applied through motivating virtual games that incorporate relevant functional tasks [26]. Aran et al. [27] found that 10 weeks of VR-based intervention enhanced cognitive function in children with unilateral CP, and they recommended the use of VR technology to optimize praxis and visuomotor and visual perceptual skills as an extension of cognitive rehabilitation. Improvement in the quality of reaching [28] and high compliance added to the advantages of the use of VR in CP management [29].

Virtual environments and wearable haptic devices can be viable alternatives to conventional therapy for improving upper extremity function in children with neuromotor impairments [30-32]. Although numerous studies investigated the use of VR technology (audio-visual feedback) [14,15,18-20], studies on the use of haptic feedback (tactile/force feedback) along with VR for children with CP remain scarce, and the research is still in the nascent stage.

Larger clinical trials have been recommended to establish the efficacy of intervention using VR approaches in specific homogenous target populations and to formulate VR training parameters that would allow optimal transfer of skills to improve function in the daily routine of patients [33]. According to a study published in 2021 by Fandim et al. [34], evidence of the benefit of VR use in CP is limited. However, future investigations may improve this.

Types of VR-based systems

The three broad categories based on the type of human-computer interactions include feedback-focused, gesture-based, and haptic-based VR systems [35]. Haptic feedback or force feedback in the virtual environment provides the required sensory input in the form of somatosensory (tactile and proprioceptive) stimulation that facilitates an adaptive motor response. A published study protocol evaluated the improvement in the smoothness of movement after intervention with haptic feedback in VR for children with dystonic CP [36]. Haptic feedback has presented better results than other VR forms in patients with chronic stroke undergoing VR-based neurorehabilitation [37].

Commercially available headsets for immersive VR experiences such as Oculus Quest (Meta Platforms, Inc., Menlo Park, CA, USA) and Vive (HTC Corporation, Xindian, New Taipei, Taiwan) are not recommended by the manufacturers for children younger than 12 years of age. Therefore, non-immersive VR with haptic feedback can be explored as a feasible option for improvement in hand function and in functional independence in young children with CP. Wii (Nintendo, Inc., Redmond, WA, USA) interactive video games may be offered as an effective supplement to conventional therapy and neurodevelopmental treatment in the rehabilitation of children with CP [38,39]. With Wii training, caregivers perceived that the children used their hands more [40]. In a study conducted on children with hemiplegic CP by Kassee et al. [41], improvement in the Melbourne Assessment (MA2) for unilateral upper extremity function was documented, along with high compliance of participants due to the motivation involved for playing these games. They suggested that intervention with Wii is a feasible intervention for home-based training. Another study likewise reported that Wii gaming was better enjoyed by children with hemiplegic CP in multiplayer version than in solo play [42]. Furthermore, Wii training promoted paretic upper extremity function, as well as improved bilateral coordination ability, which was clinically evident in children with hemiplegic CP [43]. Wii-based therapy helped improve not only impairment but also the functional level of an adolescent with diplegic CP [44]. Nevertheless, significant benefits for upper limb function remain to be seen in larger trials [45].

VR gaming has been shown to enhance the active participation of children with hemiplegic CP in upper extremity activities [46]. Error augmentation or augmented feedback in immersive VR games helped enhance BIM symmetry in children with unilateral CP [47]. PlayStation (Sony Interactive Entertainment Inc., Minato, Tokyo, Japan) is also a motivational training tool for children with CP and has the potential to improve hand function [48]. Further, the use of an advanced version of PlayStation with VR and haptic feedback-based gaming in larger sample size is recommended. Audio-visual feedback with conventional physiotherapy has been helpful in improving eye-hand coordination in children with unilateral spastic CP [49].

Kinect dance games on Xbox (Microsoft, Redmond, WA, USA) improved the balance of children with CP [50]. Similarly, Cheng et al. [51] attributed desirable changes in the dynamic balance of children with hemiplegic CP to non-immersive VR gaming. Hand exercises in a VR environment using the RAPAEL Smart Glove (Neofect, San Francisco, CA, USA) helped gain a high repetition of desired movements, facilitated functional use of the paretic upper extremity, and improved the quality of movements after a six-week intervention in a child with hemiplegic CP [52]. Home-based rehabilitation using Super Pop VR (Microsoft, Redmond, WA, USA) appeared to be feasible in modulating reaching kinematics and functional use of the paretic hand [53]. Gesture Xtreme technology (GestureTek, Silicon Valley, CA, USA) was used as a VR platform for school-aged children with CP, which significantly improved self-efficacy (as measured with the Canadian Occupational Performance Measure) [54] and playfulness (measured using the Test of Playfulness) [55]. The Leap Motion Controller (Ultraleap, Mountain View, CA, USA) successfully improved manual dexterity of the paretic hand [20]. As a variety of VR-based games have helped as adjuncts, newer available options must also be explored.

Although various studies have demonstrated that VR is effective in improving balance and gross motor function in children with CP, the extent of the usefulness of VR games in improving hand function and optimal implementation strategies in CP is, still, largely unclear [56]. Future research in the form of good-quality randomized controlled trials is required to obtain conclusive findings on the therapeutic effects of VR-based serious games on the motor skills of children with CP [57]. The findings of studies that focussed on investigating the effect of VR-based intervention on upper extremity function in children with hemiplegic CP are summarized in Table 1.

**Table 1:** Summary of main findings of the studies that focussed on investigating the effect of VR-based intervention on upper extremity function in children with hemiplegic CP

Serial number	Authors	Year	Findings related to the effect of VR on upper extremity function in children with hemiplegic CP
1.	You et al. [24]	2005	Enhanced functional motor skills including reaching, self-feeding, and dressing
2.	Golomb et al. [22]	2010	Improved hand function (reflected in functional brain changes) and forearm bone health
3.	Golomb et al. [23]	2011	14 months after the cessation of intervention, maintenance of increased hand function and forearm bone health was found
4.	Howcroft et al. [42]	2012	Increased hemiplegic limb use during play may have therapeutic advantages
5.	Pavao et al. [46]	2014	Promising tool to be incorporated into the rehabilitation process
6.	Chiu et al. [40]	2014	Did not improve hand function, though caregivers perceived that the children used their hands more
7.	Do et al. [43]	2016	Effective for enhancing motor skills of affected upper extremity and bilateral coordination ability
8.	Kassee et al. [41]	2017	Effective home-based rehabilitation strategy
9.	Shum et al. [47]	2020	Achieved more symmetrical use of hands
10.	Alwhaibi et al. [49]	2020	Improved eye-hand coordination
11.	Mirich et al. [52]	2021	High movement repetition and improved functional use of the upper extremity

## Conclusions

As derived from the studies, the duration of VR intervention sessions varied between 30 and 60 minutes, and these VR intervention sessions were conducted over a period ranging between four and 12 weeks. Most of the reviewed randomized controlled trials had highly heterogeneous groups of participants and small sample sizes. Articles published over a duration spanning almost two decades were reviewed, but studies specifically investigating the effects of VR-based intervention on the upper extremity of children with hemiplegic CP were limited. VR systems that were used in various studies include Nintendo Wii, Playstation, Xbox, Neofect Smart Glove, Leap Motion Controller, Gesture Xtreme, and Super Pop VR. With ever-evolving technology, VR-based games provide engaging, entertaining, and cost-effective means of high-intensity functional training that motivates children with spastic hemiplegic CP to use both upper extremities. This motivation to play VR games increases the voluntary effort to gain enhanced control over muscle recruitment and to finely tune the contractions of the hand muscles. This may facilitate the use of the affected upper extremity in activities of daily living, as well, which, in turn, may lead to improved functional independence and decreased burden of care on caregivers. Although the initial results are promising, further investigation on the dosimetry and effectiveness of the intervention on function in children with specific types of CP using technologically advanced VR systems is warranted.
